# TNF-α promotes human antibody-mediated complement-dependent cytotoxicity of porcine endothelial cells through downregulating P38-mediated Occludin expression

**DOI:** 10.1186/s12964-019-0386-7

**Published:** 2019-07-15

**Authors:** Hanchao Gao, Mengtao Cao, Pengfei Chen, David K. C. Cooper, Yanli Zhao, Ling Wei, Jia Xu, Zhiming Cai, Changchun Zeng, Shaodong Luan, Lisha Mou

**Affiliations:** 1 Department of Nephrology, Shenzhen Longhua District Central Hospital, Guangdong Medical University, Shenzhen, China; 2 Shenzhen Xenotransplantation Medical Engineering Research and Development Center, Institute of Translational Medicine, Shenzhen University Health Science Center, Shenzhen University School of Medicine, First Affiliated Hospital of Shenzhen University, Shenzhen Second People’s Hospital, Shenzhen, China; 3 Department of medical labrotary, Shenzhen Longhua District Central Hospital, Guangdong Medical University, Shenzhen, China; 4 Department of Surgery, Xenotransplantation Program, University of Alabama at Birmingham, Birmingham, USA

**Keywords:** Antibody-mediated complement-dependent cytotoxicity, P38, Porcine endothelial cells, TNF-α, Xenotransplantation

## Abstract

**Background:**

The major limitation of organ transplantation is the shortage of available organs. Xenotransplantation is considered to be an effective way to resolve the problem. Immune rejection is a major hurdle for the successful survival of pig xenografts in primate recipients. Cytokines play important roles in inflammation and many diseases including allotransplantation, however, their roles in xenotransplantation have been less well investigated.

**Methods:**

We assessed the role of several cytokines in xenotransplantation using an in vitro model of human antibody-mediated complement-dependent cytotoxicity (CDC). Porcine aortic endothelial cells (PAECs) and porcine iliac endothelial cells (PIECs) were selected as target cells. The complement regulators (CD46, CD55 and CD59) and junction protein genes were assessed by real-time PCR, flow cytometry, or western-blotting assay. Flow cytometry assay was also used to evaluate C3 and C5b-9 deposition, as well as the extent of human IgM and IgG binding to PIECs. Gene silencing was used to reduce genes expression in PIECs. Gene overexpression was mediated by adenovirus or retrovirus.

**Results:**

Recombinant human TNF-α increased the cytotoxicity of PAECs and PIECs in a human antibody-mediated CDC model. Unexpectedly, we found that the expression of complement regulators (CD46, CD55 and CD59) increased in PIECs exposed to human TNF-α. Human TNF-α did not modify C3 or C5b-9 deposition on PIECs. The extent of human IgM and IgG binding to PIECs was not affected by human TNF-α. Human TNF-α decreased the expression of Occludin in PIECs. Gene silencing and overexpression assay suggested that Occludin was required for human TNF-α-mediated cytotoxicity of PIECs in this model. P38 gene silencing or inhibition of P38 signaling pathway with a specific inhibitor, SB203580, inhibited the reduction of Occludin expression induced by TNF-α, and suppressed TNF-α-augmented cytotoxicity of PIECs.

**Conclusion:**

Our data suggest that human TNF-α increases the cytotoxicity of porcine endothelial cells in a human antibody-mediated CDC model by downregulating P38-dependent Occludin expression. Pharmacologic blockade of TNF-α is likely to increase xenograft survival in pig-to-primate organ xenotransplantation.

**Graphical abstract:**

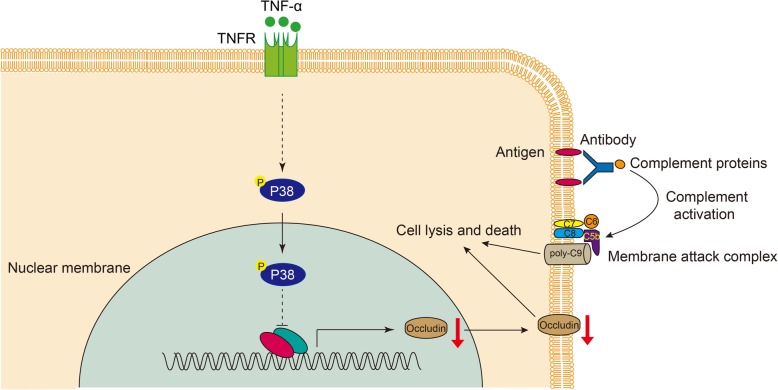

**Electronic supplementary material:**

The online version of this article (10.1186/s12964-019-0386-7) contains supplementary material, which is available to authorized users.

## Background

Organ transplantation is an effective therapy for patients with end-stage organ failure [1]. However, the major limitation of organ transplantation is the shortage of human donor organs. Xenotransplantation is considered to be a promising way to solve the problem [2]. Pigs are considered to be the most suitable source of organs for xenotransplantation, according to physiological, anatomical, economic, and ethical considerations [3]. However, immune rejection is a major obstacle after pig organ transplantation in primates [4].

In pig-to-human xenotransplantation, porcine vascular endothelial cells (ECs) are the first defense. They interact with human immune cells, and are activated by cytokines or chemokines produced by the human immune cells [5]. Porcine ECs are also the first cells to be attacked by the recipient immune system in xenotransplantation. ECs dysfunction and injury are critical factors in promoting inflammation and coagulation, which decrease the survival of the pig xenograft [6, 7].

The complement system consists of several tightly-regulated proteins that play important roles in the inflammatory response and in host defense [8, 9]. Three different pathways (alternative, classical, and lectin) can activate the complement system. The pathways converge at C3. Recipient antibodies bind to the pig xenoantigens on the vascular ECs, and activate the complement system, inducing antibody-mediated complement-dependent cytotoxicity (CDC). When complement is activated by antibodies, activated complement fragments and complexes transmit the stimulatory signals, and result in the formation of the membrane attack complex (MAC) which consists of the C5b, C6, C7, C8, and C9 complement proteins (C5b-9). The MAC inserts into the plasma membrane and leads to the cell death [8, 9].

In pig-to-primate organ transplantation, cytokines and chemokines are secreted by recipient cells [10]. Previously, we reported that human angiopoietin-1 (Ang-1) and Ang-2 protected porcine ECs from human antibody-mediated CDC by activating phosphatidylinositide 3-kinase (PI3K)/AKT pathway [11]. However, EGF, bFGF, VEGF, IL-33, and IL-17 did not affect the cytotoxicity of porcine ECs. Human IL-4 and IL-13 also mediated protection of porcine ECs [12]. However, the roles of other important pro-inflammatory cytokines or chemokines, such as IL-8, IL-6, TNF-α, and G-CSF, in human antibody-mediated CDC models have not been fully investigated.

In order to explore this topic, we chose eight human cytokines (IL-2, IL-15, IL-8, IL-6, TNF-α, G-CSF, G-MCSF and IFNγ) and evaluated their effects in a human antibody-mediated CDC model. Porcine ECs were chosen as the target cells. We demonstrated that TNF-α promoted human antibody-mediated CDC of porcine ECs and was dependent on P38-mediated reduction of Occludin expression. This provides a possible explanation for the pathological role of TNF-α in xenotransplantation.

## Methods

### Reagents and cell culture

Recombinant human (rh) IL-2, IL-15, IL-8_,_ IL-6, TNF-α, G-CSF, G-MCSF, IFNγ, and IL-4 were purchased from R&D Systems (Minneapolis, MN, USA). FITC-conjugated goat anti-human IgM, IgG or isotype-matched antibodies were purchased from Invitrogen (Carlsbad, CA, USA). FITC-conjugated goat anti-mouse IgG was obtained from Jackson ImmunoReasearch Laboratories (West Grove, PA, USA). Anti-actin, anti-pJNK, anti-JNK, anti-pP38, anti-P38, anti-ERK and anti-Cleaved Caspase 3 were purchased from Cell Signaling Technology (Boston, MA, USA); anti-pERK from Santa Cruz Biotechnology (Shanghai, China); anti-CD46 from Bio-rad (Hercules, CA, USA); anti-CD55 from LifeSpan BioSciences (Seattle, WA, USA); anti-Claudin 2, anti-C3 (Alexa Flour® 488), anti-C3c (FITC) and anti-C5b-9 from Abcam (Shanghai, China); anti-Occludin and anti-Zo 1 from Thermo Fisher Scientific (Rockford, IL, USA); SB203580, SP600125 and PD98059 from Selleck (Shanghai, China); Cell Counting Kit-8 (CCK8) from Dojindo Laboratories (Kumamoto, Japan); and Neutral Red from Sangon Biotech (Shanghai, China).

HEK293 cells were maintained in DMEM supplemented with 10% (vol/vol) FBS, 1% (vol/vol) penicillin G/streptomycin (P/S) at 37 °C with 5% CO_2_. Porcine iliac endothelial cells (PIECs) were purchased from the Type Culture Collection of the Chinese Academy of Sciences; the cells were cultured with RPMI-1640 containing 10% (vol/vol) FBS, 1% (vol/vol) P/S at 37 °C with 5% CO_2._ Porcine aortic endothelial cells (PAECs) were isolated from one Chinese Wuzhishan wild-type pig, and cultured as previously described [13].

### Human antibody-mediated complement-dependent cytotoxicity

PIECs or PAECs (4 × 10^3^) were seeded into 96-well plates and treated with recombinant cytokines for 0, 24 h, 48 h, or 72 h. The supernatant was removed and replaced with RPMI-1640, containing 20% pooled human serum (experimental group-the serum was pooled from several healthy volunteers [*n* = 20], including all ABO blood types), or 20% heat-inactivated human serum (control group) for 2 h. After 2 h, CCK8 was used to assess the viability of PIECs or PAECs. The supernatant was removed and replaced with RPMI-1640 containing 10% CCK8 for 2 h. Two hours later, the absorbance values of wells were measured with OD 450 that was read using a multiscan GO spectrophotometer (Thermo Fisher). The percentage cell death (cytotoxicity) was calculated by the following formula:

$$ \%\mathrm{cytotoxicity}=\left(\mathrm{OD}\ \mathrm{of}\ \mathrm{control}\ \mathrm{group}-\mathrm{OD}\ \mathrm{of}\ \mathrm{experimental}\ \mathrm{group}\right)/\mathrm{OD}\ \mathrm{of}\ \mathrm{control}\ \mathrm{group}\times 100. $$

**Fig. 1:**
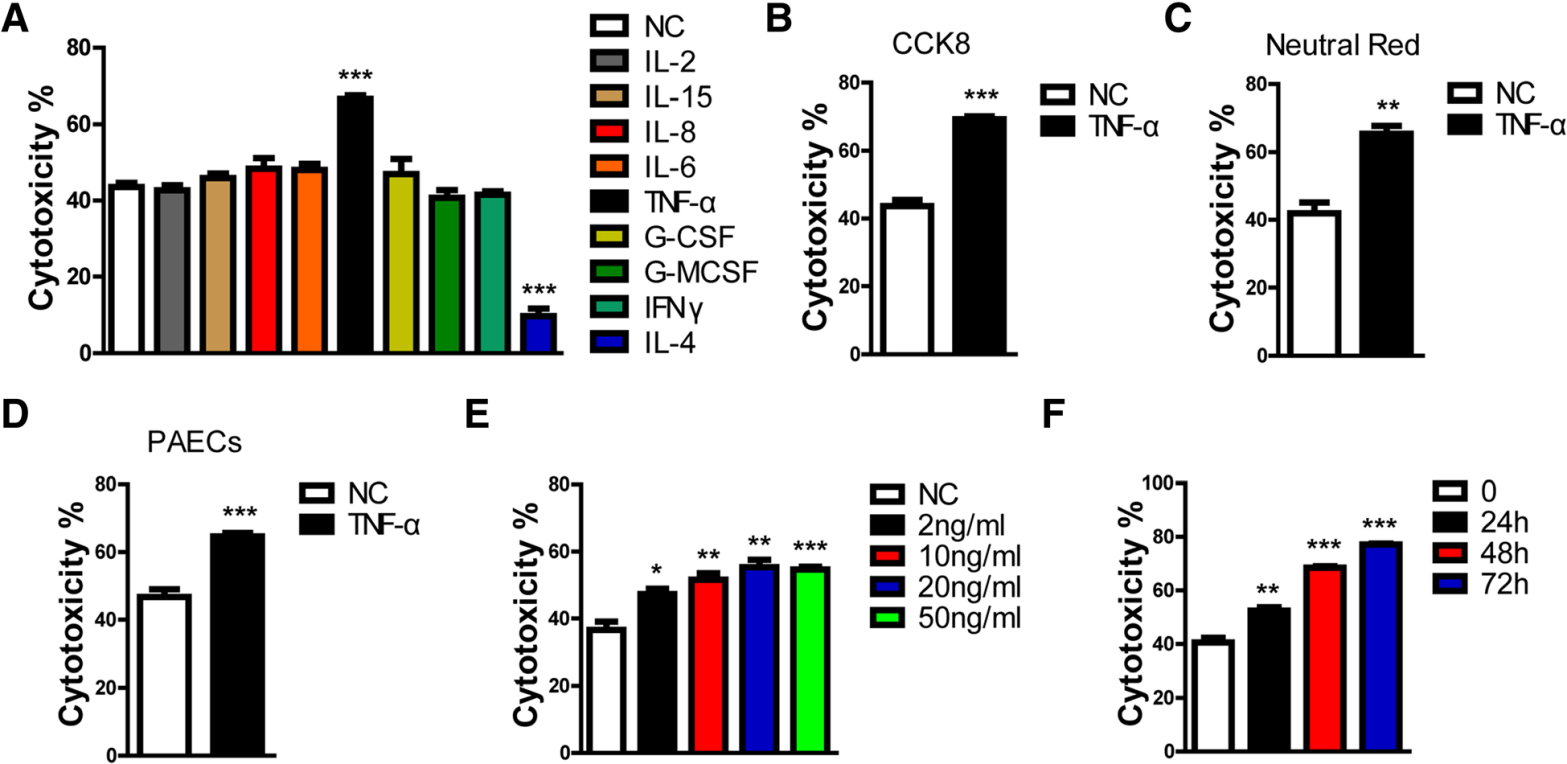
TNF-α promoted the cytotoxicity of porcine ECs in a human antibody-mediated complement-dependent cytotoxicity (CDC) model. **a** PIECs were treated with recombinant human IL-2 (20 ng/ml), IL-15 (100 ng/ml), IL-8 (100 ng/ml), IL-6 (20 ng/ml), TNF-α (20 ng/ml), G-CSF (100 ng/ml), G-MCSF (50 ng/ml), IFNγ (50 ng/ml), IL-4 (20 ng/ml), or medium as a negative control (NC) for 48 h and then exposed to human serum to induce antibody-mediated CDC. **b-c** PIECs were treated with rhTNF-α (20 ng/ml), or medium as a NC for 48 h and then exposed to human serum to induce antibody-mediated CDC. The extent of cytotoxicity was assessed by Cell Counting Kit-8 (CCK8) (**b**) or neutral red (**c**). **d** PAECs were treated with rhTNF-α (20 ng/ml), or medium as a NC for 48 h and then exposed to human serum to induce antibody-mediated CDC. **e** PIECs were treated with different concentrations of rhTNF-α (2, 10, 20 or 50 ng/ml) or medium as a NC for 48 h and then exposed to human serum to induce antibody-mediated CDC. **f** PIECs were treated with rhTNF-α (20 ng/ml) or medium as a NC for 0, 24, 48 or 72 h, and then exposed to human serum to induce antibody-mediated CDC. Data are representative of at least three independent experiments (mean ± SEM). *p < 0.05, **p < 0.01, ***p < 0.001 by Student’s t test

Cell viability was evaluated by neutral red staining (Fig. 1c). The percentage cytotoxicity was calculated using the same formula as for CCK8.

### Adenoviral-mediated human TNF-α expression in PIECs

The construction of an adenoviral-mediated human TNF-α overexpression plasmid has been described previously [14]. After the plasmid of adenovirus (Adv)-TNFα was constructed, the recombinant plasmid Adv-TNFα or empty vector (Adv-EV) was transfected into HEK293A cells for adenovirus packaging. The recombinant adenoviruses were amplified, purified, titrated and diluted. The final concentration of adenovirus particles was 10^10^–10^11^ per ml PBS. PIECs were infected with Adv-TNFα or Adv-EV for 72 h, and then exposed to human serum to induce antibody-mediated CDC.

### Real time-PCR

The technique of real-time PCR has been reported previously [15, 16]. Briefly, total RNA of PIECs was extracted with TRIzol® Reagent (Invitrogen). The cDNA samples were synthesized with Transcript First-Strand cDNA Synthesis SuperMix (TransGene Biotech, Beijing, China). The levels of the genes of interest were quantified using SYBR *Premix Ex Taq* kit (Takara Bio, Dalian, Liaoning, China). The expression levels of the genes were calculated by the 2^-ΔΔCt^ method and normalized to pig glyceraldehyde-3-phosphate dehydrogenase (GAPDH). Amplification of cDNA was performed on a ViiA 7 Real-Time PCR system (Applied Biosystems, Foster City, CA, USA) with the sequences of oligonucleotide primers shown in Additional file 1: Table S1.

### Enzyme-linked immunosorbent assay (ELISA)

After collecting supernatant of PIECs infected with Adv-EV or Adv-TNFα for 72 h, the protein levels of human TNFα in the supernatant were measured with TNF-α ELISA kits (Abcam), according to the manufacturer’s instructions.

### Gene knockdown

The two sequences for porcine Occludin gene knockdown were 5′- ggaagactggatcagggaata-3′ and 5′-gcaggagtacaagagcttaca-3′. The two sequences for porcine Zo 1 gene were 5′-ggatggcgctacaagtgatga-3′ and 5′-ggactcctctggaatgcatca-3′. The two sequences for porcine Claudin 2 gene knockdown were 5′-tctcctcgttggcctgtat-3′ and 5′-gcatcatttcctccctgtt-3′. The scrambled control siRNA sequence was 5′-ggatccttgacaataccaa-3′. The siRNA double-stranded oligonucleotides sequences were cloned into pLSLG lentiviral vector. The respective lentiviral vectors and packaging vectors were transfected into HEK 293FT cells. After 2 days, the virus was collected and PIECs were infected. Four days later, the infected cells were used for experiments as described.

The two sequences for porcine P38 gene knockdown were 5′-ggcaagaaactacattcaa-3′ and 5′-ggcccggcatacagatgat-3′. The siP38 oligos and control oligo were transfected into PIECs using jetPrime® DNA and siRNA Transfection Reagent (Polyplus transfection, Illkirch, France) according to the instructions. After 48 h, the transfected cells were used for experiments.

### Retrovirus-mediated Occludin expression

Occludin was cloned using a pMSCV-IRES-GFP vector. Retrovirus vectors and helper vectors were transfected into HEK293FT cells. After 2 days, virus was collected for infection of PIECs. At day 4 after infection, cells were used for experiments.

### Flow cytometry analysis

Human IgM and IgG binding using flow cytometry was performed as previously described [17, 18]. In brief, pooled human serum from several healthy volunteers or human serum minus IgG, IgA and IgM (from Sigma, St. Louis, MO, USA) was heat-inactivated at 56 °C for 30 min. PIECs (1 × 10^6^) were incubated with 20% heat-inactivated human serum (HIHS) or heat-inactivatd human serum minus IgG, IgA and IgM (HIHS[−]) for 30 min at 37 °C. After washing with PBS, 10% goat serum was used to prevent non-specific binding. Then the cells were incubated with FITC-conjugated goat anti-human IgM or IgG antibody for 30 min at 4 °C in the dark. Secondary antibody only, without serum, acted as a control.

For CD46 and CD55 expression, and C5b-9 deposition, PIECs (1 × 10^6^) were incubated with 20% human serum for 30 min at 37 °C. Then the cells were incubated with anti-CD46, anti-CD55, or C5b-9 antibody for 1 h at 4 °C in the dark. Isotype antibody acted as a control. After washing, the cells were incubated with FITC-conjugated goat anti-mouse IgG for 30 min at 4 °C in the dark.

For C3 deposition, PIECs (1 × 10^6^) were incubated with 20% human serum for 30 min at 37 °C. Then the cells were incubated with anti-C3 (Alexa Flour® 488) or anti-C3c (FITC) antibody for 1 h at 4 °C in the dark. Corresponding isotype antibodies acted as controls.

After washing, the treated cells were analyzed by flow cytometry using BD FACSAria II (BD Bioscience, San Jose, CA, USA). The degree of antibody binding was assessed by geometric mean fluorescence intensity (Gmean).

### Western-blotting analysis

The procedure of western-blotting analysis has been previously reported [19]. In brief, PIECs were harvested after washing with ice-cold PBS, and lysed for 30 min in ice-cold RIPA lysis buffer supplemented with 10 mM sodium fluoride (NaF), 1 mM Na_3_VO_4_, 1 mM phenylmethylsulfonyl fluoride, and protease inhibitor cocktail (Roche, Indianapolis, IN, USA). The protein concentration was assessed by BCA Protein Assay Kit (Thermo Fisher) according to the instructions. Every protein sample (20 μg) was separated by 10% SDS-PAGE. After the proteins were transferred onto polyvinylidene fluoride (PVDF) membranes (Millipore, Billerica, MA, USA), the PVDF membranes were blocked with 5% skim milk at room temperature for 1 h and then were incubated with primary antibody overnight at 4 °C. After incubation with secondary antibody for 1 h at room temperature, the blots were visualized with enhanced chemiluminescence (ECL) detection reagents (Millipore).

### Permeability assay

Cells were grown on 3 μm pore Transwell filters (Corning) until confluent. After the cells were washed twice with PBS, 100 μg/ml fluorescein isothiocyanate (FITC)-conjugated 40 kDa dextran (Sigma) was added to the apical compartment for 30 min. The fluorescence of basolateral medium was measured using a spectrofluorometer (Thermo Fisher) at an excitation wavelength of 492 nm and detection wavelength of 520 nm.

### Statistical analysis

Experimental data are presented as the mean ± SEM using Prism software (GraphPad software). Statistical significance between the groups was calculated by using a two-tailed Student’s t test (Microsoft Office Excel software). *p* values < 0.05 were considered significant.

## Results

### Human TNF-α promotes the cytotoxicity of porcine ECs in a human antibody-mediated CDC model

Previously, we found that recombinant human angiopoietin-1 (rhAng-1) and rhAng-2 protected PIECs from human antibody-mediated CDC [11]. Human IL-4 and IL-13 also protected porcine ECs in this model [12]. Besides these cytokines, we asked whether other human cytokines also play roles in this model.

PIECs were treated with recombinant human TNF-α, IL-2, IL-15, IL-8, IL-6, G-CSF, G-MCSF, IFNγ, IL-4 or medium for 48 h, and then exposed to human serum (heat-inactivated serum as a control) to induce antibody-mediated CDC. As expected, we found that human TNF-α increased the cytotoxicity of PIECs in the human antibody-mediated CDC model. Cytotoxicity (by CCK8) increased from 43 to 66% (Fig. 1a). However, other cytokines, such as human IL-2, IL-15, IL-8, IL-6, G-CSF, G-MCSF, and IFNγ, had no effect on the cytotoxicity of PIECs (Fig. 1a). Human IL-4, as a positive control, protected PIECs from human antibody-mediated CDC which was consistent with a previous report (Fig. 1a) [12]. To further confirm our results, both CCK8 and neutral red were used to assess the cytotoxicity, and we obtained similar results (Fig. 1b-c).

**Fig. 2:**
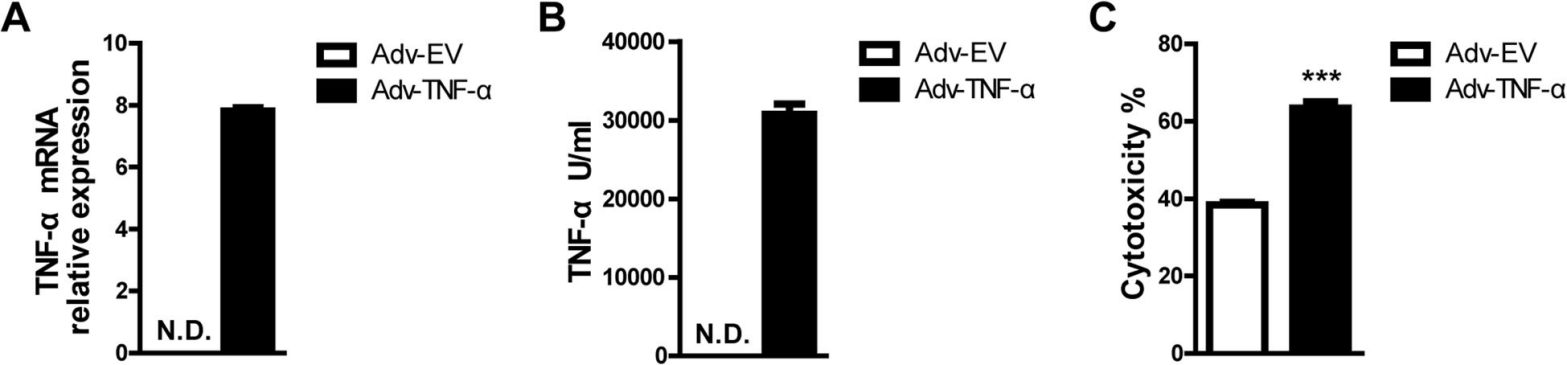
Human TNF-α ectopically expressed in PIECs (using an adenoviral system) augmented the cytotoxicity of PIECs in a human antibody-mediated CDC model. **a** The plasmid which expressed the human TNF-α gene was constructed using an adenovirus (Adv) system. The Adv was packaged. PIECs were infected with Adv-TNF-α, or Adv-EV (empty vector) for 72 h. Total RNA was collected for RT-PCR analysis. The mRNA level was assessed with a human TNF-α primer, and normalized to pig GAPDH. **b** PIECs were infected with Adv-TNF-α, or Adv-EV for 72 h. The supernatant was collected for enzyme-linked immunosorbent assay (ELISA). **c** PIECs were infected with Adv-TNF-α, or Adv-EV for 72 h, and then exposed to human serum to induce antibody-mediated CDC. Data are representative of at least three independent experiments (mean ± SEM). ***p < 0.001 by Student’s t test. N.D. = not determined

Because PIECs are immortal cell lines, we further evaluated the role of TNF-α in primary porcine aortic endothelial cells (PAECs) and found that rhTNF-α also promoted the cytotoxicity of PAECs (Fig. 1d). Additionally, we found that rhTNF-α promoted the cytotoxicity of PIECs in a dose-dependent manner and time-dependent manner (Fig. 1e-f). We ectopically expressed human TNF-α in PIECs with an adenoviral system. The real-time PCR assay and ELISA assay suggested that both the mRNA and protein levels of human TNF-α were greatly overexpressed in PIECs infected with Adv-TNF-α (Fig. 2a-b). PIECs infected with Adv-TNF-α significantly promoted the cytotoxicity of PIECs in the human antibody-mediated CDC model (Fig. 2c). Collectively, these data suggest that human TNF-α promotes human antibody-mediated CDC of porcine ECs.

### Human TNF-α increases the expression of complement regulators but does not affect complement deposition or human antibody binding to PIECs

Since TNF-α could induce cell apoptosis in several cells directly, we wanted to know whether TNF-α caused PIEC apoptosis. We found that TNF-α alone did not induce cleaved caspase 3 in PIECs exposed to TNF-α for 48 h (Additional file 2: Figure S1A). We also found that TNF-α did not affect the cell proliferation of PIECs exposed to TNF-α for 24 h, 48 h, or 72 h, and even slightly increased the cell number of PIECs exposed to TNF-α for 96 h (Additional file 2: Figure S1B). Moreover, IL-2, IL-15, IL-8, IL-6, G-CSF, G-MCSF, IFNγ did not affect the cell number of PIECs exposed to the corresponding cytokine for 48 h (Additional file 2: Figure S1C). However, IL-4 obviously increased the cell number of PIECs treated by IL-4 for 48 h (Additional file 2: Figure S1C). The data suggested that TNF-α did not cause PIECs death directly.

**Fig. 3:**
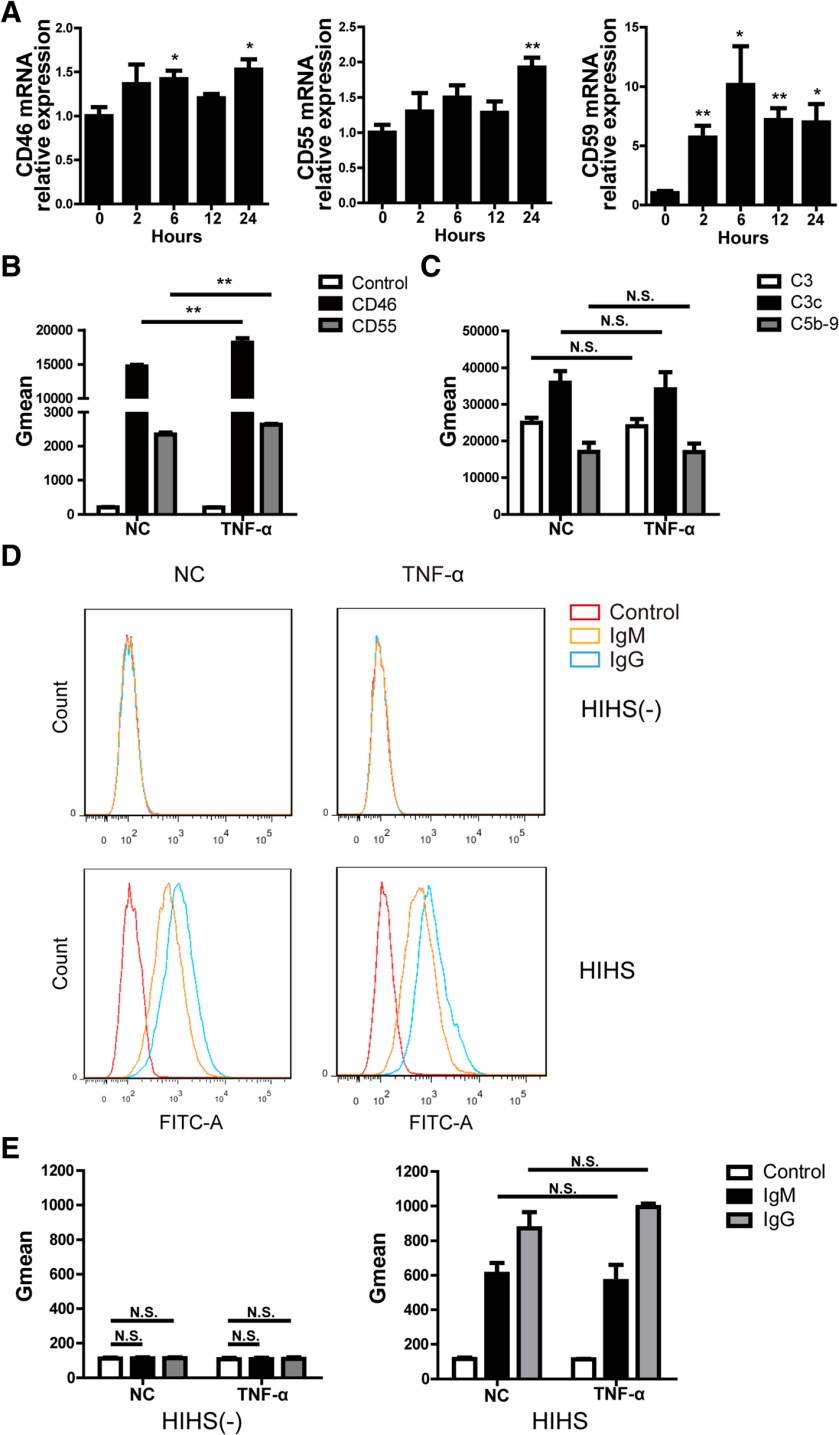
Expression of complement-regulatory proteins on PIECs was increased, and the extent of human IgG and IgM binding to PIECs, and C3 or C5b-9 deposition on PIECs was not affected by rhTNF-α. **a** PIECs were treated with rhTNF-α (20 ng/ml) for 0, 2, 6 or 12 h. The mRNA levels of CD46, CD55, or CD59 were measured by RT-PCR. **b-c** PIECs were treated with rhTNF-α (20 ng/ml), or medium as a NC for 48 h, and then incubated with human serum for 30 min. The expression of CD46 (**b**), CD55 (**b**), C3 deposition (C3 and C3c) (**c**) or C5b-9 deposition (**c**) was assessed by flow cytometry. The quantitation data were presented by geometric mean fluorescence intensity (Gmean). **d-e** PIECs were treated with rhTNF-α (20 ng/ml), or medium as a NC for 48 h, and then incubated with heat-inactivated human serum (HIHS) or heat-inactivatd human serum minus IgG, IgA and IgM (HIHS[−]) for 30 min. Human IgM or IgG binding to PIECs was measured by flow cytometry (**d**). The extent of human IgM or IgG binding to PIECs was evaluated by Gmean (**e**). Data are representative of at least three independent experiments (mean ± SEM). *p < 0.05, **p < 0.01 by Student’s t test. N.S. = no significance

As is known, complement regulators (CD46, CD55 and CD59) suppress the activation of complement and subsequently reduce CDC [20, 21]. We asked whether human TNF-α promoted the killing of PIECs through decreasing the expression of complement regulators. Unexpectedly, we found that TNF-α increased the mRNA level of CD46, CD55 and CD59 (Fig. 3a). We also checked the protein level of CD46 and CD55 by flow cytometry and found that CD46 and CD55 were upregulated by TNF-α in the human antibody-mediated CDC model (Fig. 3b). The data demonstrated that changes in expression of complement regulators was not a factor causing TNF-α to promote the killing of PIECs.

Next, we asked whether TNF-α affected complement activation. We assessed C3 (with anti-C3 and anti-C3c antibodies) and C5b-9 deposition after the PIECs were incubated with human serum and found that TNF-α did not affect C3 or C5b-9 deposition on PIECs (Fig. 3c). The results suggested that the increase killing of PIECs by TNF-α might not be due to complement activation or membrane attack complex (MAC) formation.

We also checked the binding of human antibody to PIECs. The binding of IgG or IgM to PIECs increased when the PIECs were exposed to heat-inactivated human serum (HIHS) but not heat-inactivatd human serum minus IgG, IgA and IgM (HIHS[−]) (Fig. 3d-e). However, TNF-α did not increase the binding of IgG or IgM with PIECs (exposed to HIHS) compared to negative control group (Fig. 3d-e).

Together, these data demonstrate that complement regulators, complement activation and antibody binding are not the primary factors in the TNF-α promotion of cytotoxicity of PIECs.

### Occludin is required for TNF-α promoting the cytotoxicity of PIECs in human antibody-mediated CDC model

**Fig. 4:**
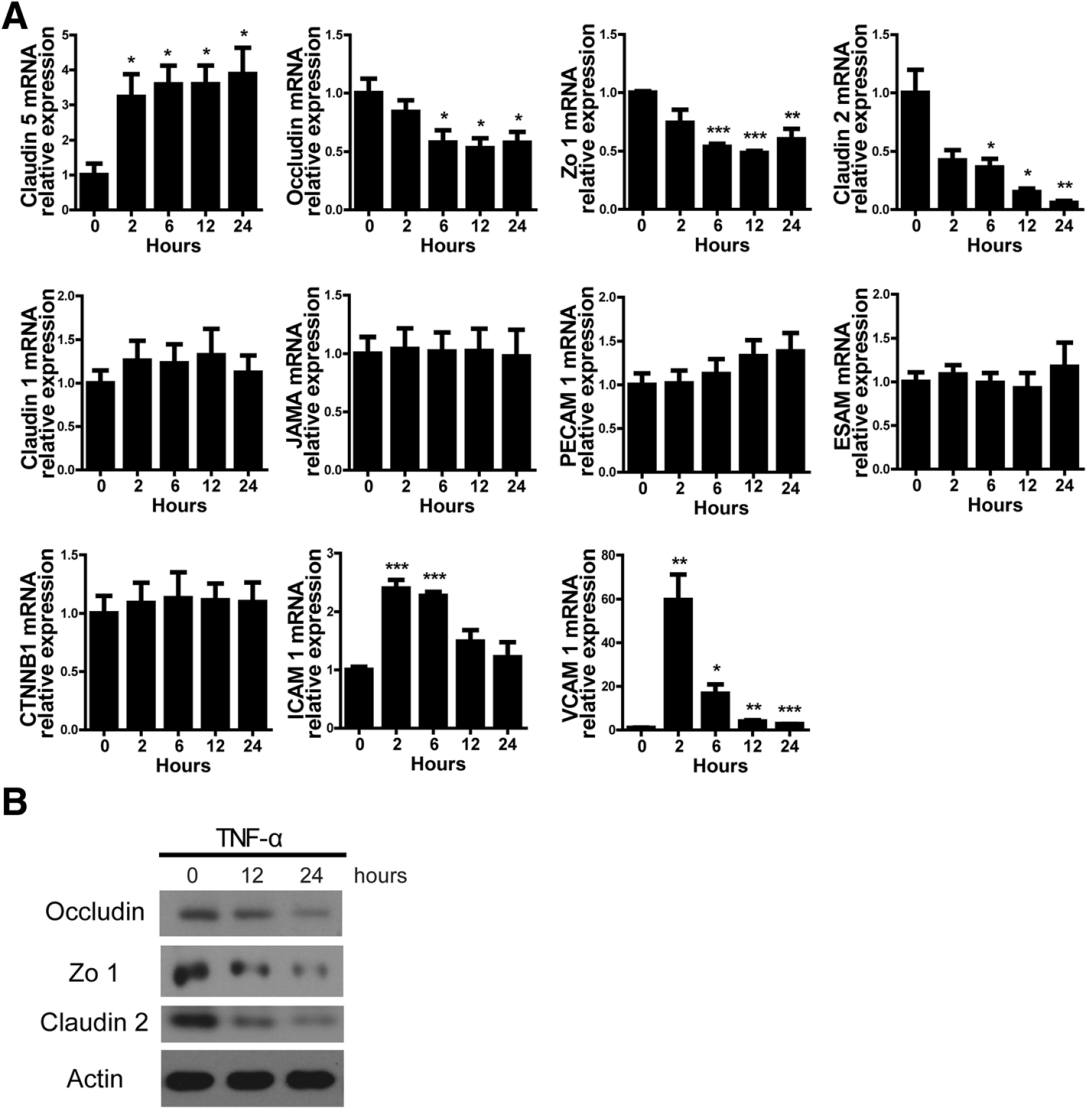
The expression of Zo 1, Claudin 2, or Occludin was downregulated in PIECs exposed to TNF-α. **a** PIECs were treated with rhTNF-α (20 ng/ml) for 0, 2, 6, 12 or 24 h. The mRNA levels of indicated genes were measured by RT-PCR. **b** PIECs were treated with rhTNF-α (20 ng/ml) for 0, 12, or 24 h. Lysates were analyzed by western-blotting with antibodies against Occludin, Zo 1, Claudin 2, and actin. Data are representative of at least three independent experiments (mean ± SEM). *p < 0.05, **p < 0.01, ***p < 0.001 by Student’s t test

A previous report suggested that IL-4 increased the expression of the junction gene Claudin 5 to mediate the protection of porcine ECs from human antibody-mediated CDC [22]. We wanted to check whether TNF-α regulates the expression of junction genes to increase the killing of PIECs. Real-time PCR analysis suggested that TNF-α increased the mRNA level of Claudin 5, and reduced the mRNA level of Occludin, Zo 1, or Claudin 2 in PIECs (Fig. 4a). ICAM1 and VCAM1 as positive controls were induced in PIECs treated by TNF-α, which was consistent with our previous report [15]. The mRNA level of other junction genes (Claudin 1, JAMA, PECAM 1, ESAM, and CTNNB1) in PIECs was not regulated by TNF-α (Fig. 4a). The protein level of Occludin, Zo 1 and Claudin 2 was also obviously decreased in PIECs exposed to TNF-α (Fig. 4b).

**Fig. 5:**
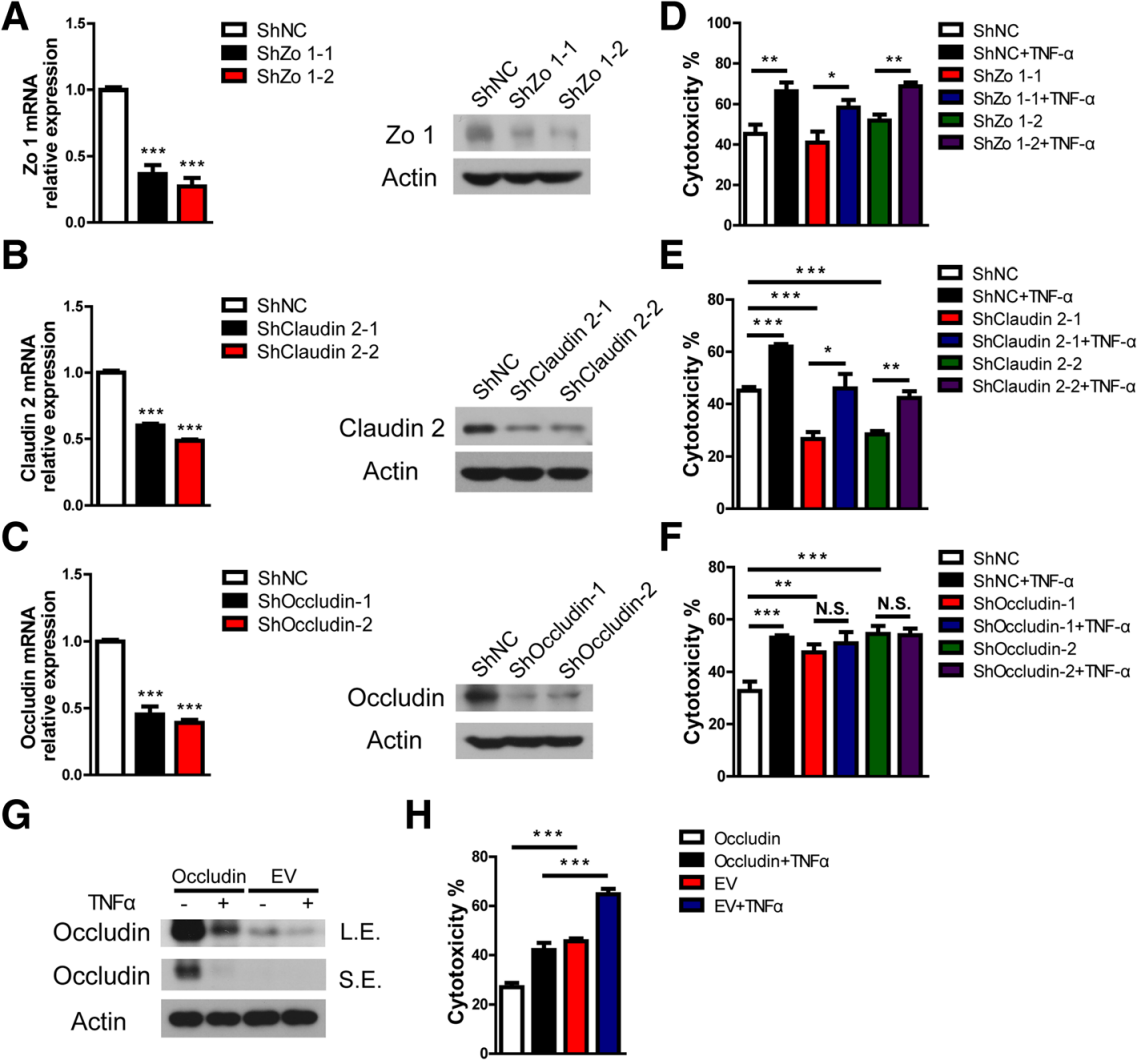
In a human antibody-mediated CDC model, TNF-α promoted the cytotoxicity of PIECs and was dependent on Occludin. **a-c** PIECs were infected with control lentivirus or with lentivirus expressing Zo 1- (**a**), Claudin 2- (**b**), or Occludin- (**c**) specific siRNA. After 4 days, total RNA was collected and the mRNA level of Zo 1 (**a**), Claudin 2 (**b**), or Occludin (**c**) was analysed by RT-PCR (left side), and lysates were analyzed by western-blotting (right side) with antibodies against Zo 1 (**a**), Claudin 2 (**b**), Occludin (**c**), or actin. **d-f** The infected cells shown in (**a-c**), which corresponded to (**d-f**) respectively, were treated with or without rhTNF-α (20 ng/ml) for 48 h, and then exposed to human serum to induce antibody-mediated CDC. **g** PIECs infected with control virus (EV) or retrovirus encoding Occludin were left untreated or treated with rhTNF-α (20 ng/ml) for 48 h. Lysates were analyzed by immunoblotting with antibodies against Occludin and actin. **h** The infected cells shown in (**g**) were treated with or without rhTNF-α (20 ng/ml) for 48 h and then exposed to human serum to induce antibody-mediated CDC. Data are representative of at least three independent experiments (mean ± SEM). *p < 0.05, **p < 0.01, ***p < 0.001 by Student’s t test. N.S. = no significance, L.E. = long exposure, S.E. = short exposure

Because upregulation of Claudin 5 suppresses human antibody-mediated CDC, we asked whether downregulation of Zo 1, Claudin 2, or Occludin promoted human antibody-mediated CDC. PIECs were infected with lentivirus encoding the corresponding siRNA to knockdown Zo 1, Claudin 2, or Occludin. The mRNA and protein levels of Zo 1, Claudin 2, or Occludin were significantly reduced in corresponding infected PIECs (Fig. 5a-c). We found that loss of Zo 1 did not affect the cytotoxicity of PIECs treated with or without TNF-α compared to respective controls (Fig. 5d). Surprisingly, loss of Claudin 2 decreased cell death compared to controls (Fig. 5e). However, TNF-α still promoted cytotoxicity in the Claudin 2 gene knockdown group (Fig. 5e). Intriguingly, we found that loss of Occludin significantly augmented cell death compared to controls (Fig. 5f). Moreover, TNF-α did not increase the cytotoxicity of Occludin-knockdown PIECs in the human antibody-mediated CDC model (Fig. 5f). These data suggest that Occludin is critical for the TNF-α promotion of cytotoxicity of PIECs.

To further confirm the result, we ectopically expressed porcine Occludin in PIECs using retrovirus-mediated gene expression (Fig. 5g). In human antibody-mediated CDC, overexpression of Occludin decreased cell death compared to respective controls treated with or without TNF-α (Fig. 5h). Collectively, these data suggest that TNF-α promotes the killing of PIECs in human antibody-mediated CDC through downregulating Occludin expression.

Since Claudin 2 promoted the killing of PIECs and Claudin 5 mediated the protection of porcine ECs in human antibody-mediated CDC, we asked whether Occludin promoted the killing of PIECs by affecting the expression of Claudin 2, Claudin 5 or other junction genes. Overexpression or gene silencing of Occludin did not affect the mRNA level of Claudin 2, Claudin 5, or other junction genes (Zo 1, Claudin 1, JAMA, PECAM1, ESAM or CTNNB1) in PIECs (Additional file 3: Figure S2A-B). Morover, we also found that loss of Claudin 2 did not affect Occludin or Claudin 5 expression in PIECs (Additional file 3: Figure S2C). The data demonstrated that Occludin protected the PIECs from cytotoxicity, and was not dependent on regulating the expression of Claudin 2, Claudin 5, or other junction genes.

### Human TNF-α promotes human antibody-mediated CDC of PIECs through the P38 signaling pathway

**Fig. 6:**
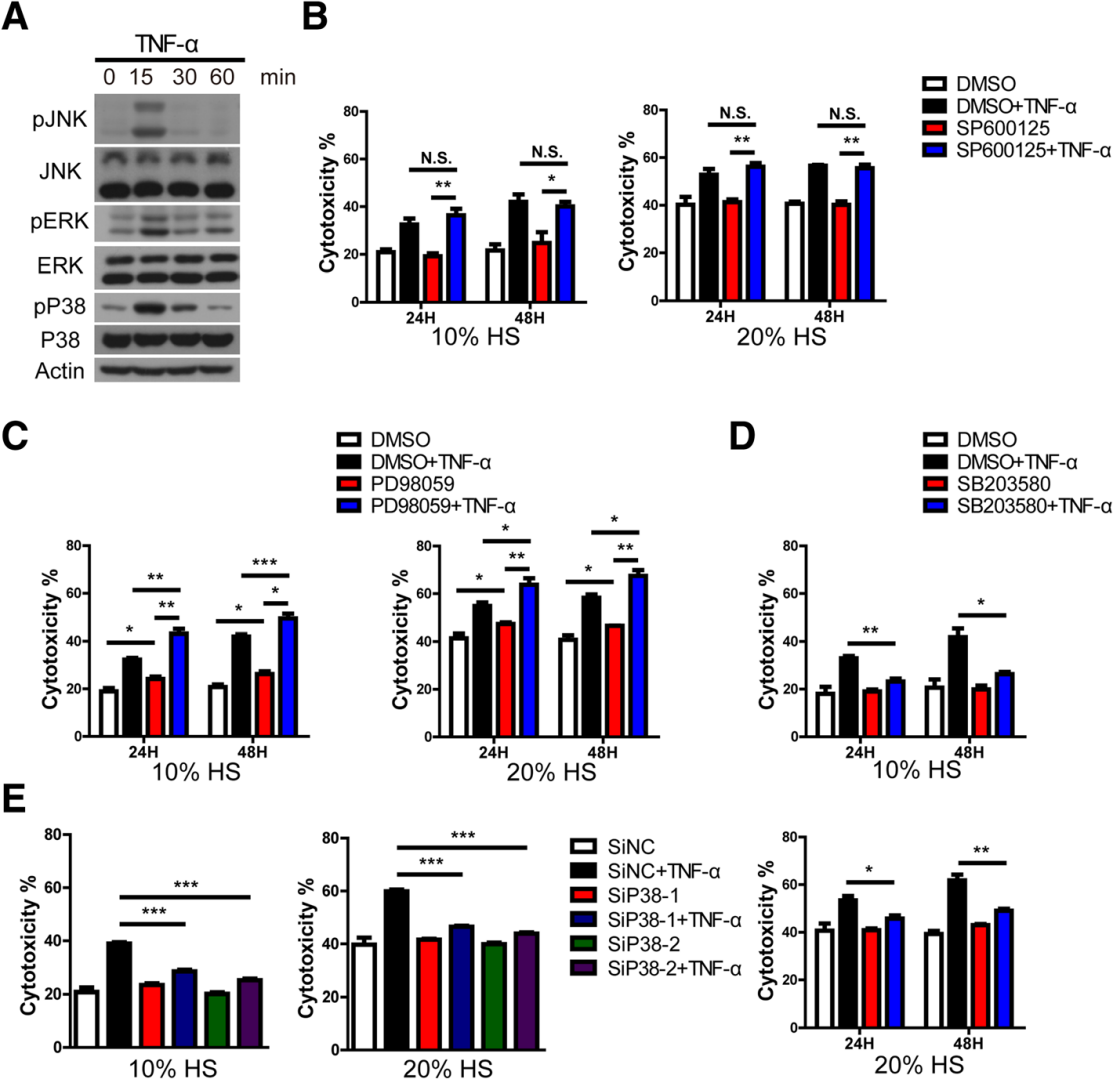
In a human antibody-mediated CDC model, SB203580 or loss of P38 suppressed the cytotoxicity of PIECs exposed to TNF-α. **a** PIECs were treated with rhTNF-α (20 ng/ml) for 0, 15, 30, 60 min. Lysates were analyzed by western blotting with antibodies against pJNK, JNK, pERK, ERK, pP38, P38 and actin. **b-d** PIECs were pretreated with DMSO, SP600125 (**b**), PD98059 (**c**) or SB203580 (**d**) for 30 min and treated with rhTNF-α (20 ng/ml) for 24 h or 48 h and then exposed to 10% or 20% human serum (HS) to induce antibody-mediated CDC. **e** The siP38 oligos and control oligo were transfected into PIECs. After 48 h, the transfected cells were treated with rhTNF-α (20 ng/ml) for 48 h and then exposed to 10% or 20% HS to induce antibody-mediated CDC. Data are representative of at least three independent experiments (mean ± SEM). *p < 0.05, **p < 0.01, ***p < 0.001 by Student’s t test. N.S. = no significance

Our previous report revealed that P38 and JNK (which belong to mitogen-activated protein kinases [MAPKs]) were activated in PAECs by human TNF-α [15]. We also found that human TNF-α activated JNK, ERK and P38 in PIECs (Fig. 6a). To determine whether TNF-α promotion of the cytotoxicity of PIECs is dependent on MAPKs in human antibody-mediated CDC, we treated PIECs with MAPKs inhibitors (JNK inhibitor SP600125, ERK inhibitor PD98059, P38 inhibitor SB203580) and then treated PIECs with TNF-α at different time points and with different human serum concentrations. Inhibition of JNK with SP600125 did not affect the TNF-α promotion of cell death of PIECs exposed to TNF-α for 24 h or 48 h with 10% serum or 20% serum (Fig. 6b). ERK inhibition using PD98059 increased cell death of PIECs (exposed to TNF-α for 24 h or 48 h with 10% serum or 20% serum) compared to the control group (Fig. 6c), which was consistent with a previous report [23]. However, TNF-α still obviously augmented the killing of PIECs treated by PD98059, irrespective of the period of exposure or serum concentration (Fig. 6c). These data suggested that JNK and ERK were not responsible for the TNF-α promotion of the killing of PIECs.

Surprisingly, we found that inhibition of P38 with SB203580 obviously suppressed TNF-α-augmented cytotoxicity of PIECs exposed to TNF-α for 24 h or 48 h with 10% serum or 20% serum (Fig. 6d). The cytotoxicity rate of PIECs treated by SB203580 plus TNF-α was obviously decreased compared to DMSO plus TNF-α group (Fig. 6d). In order to confirm the result, we knocked down the P38 expression in PIECs with siRNA oligos. The mRNA and protein levels of P38 were obviously decreased by siRNA oligos (Additional file 4: Figure S3). We also found that loss of P38 significantly decreased TNF-α-augmented cytotoxicity of PIECs exposed to TNF-α for 24 h or 48 h with 10% serum or 20% serum (Fig. 6e). Together, these data demonstrate that TNF-α promotion of cytotoxicity of PIECs is dependent on the P38 signaling pathway.

### P38 signaling is essential for the reduction of Occludin expression in PIECs treated by TNF-α

**Fig. 7:**
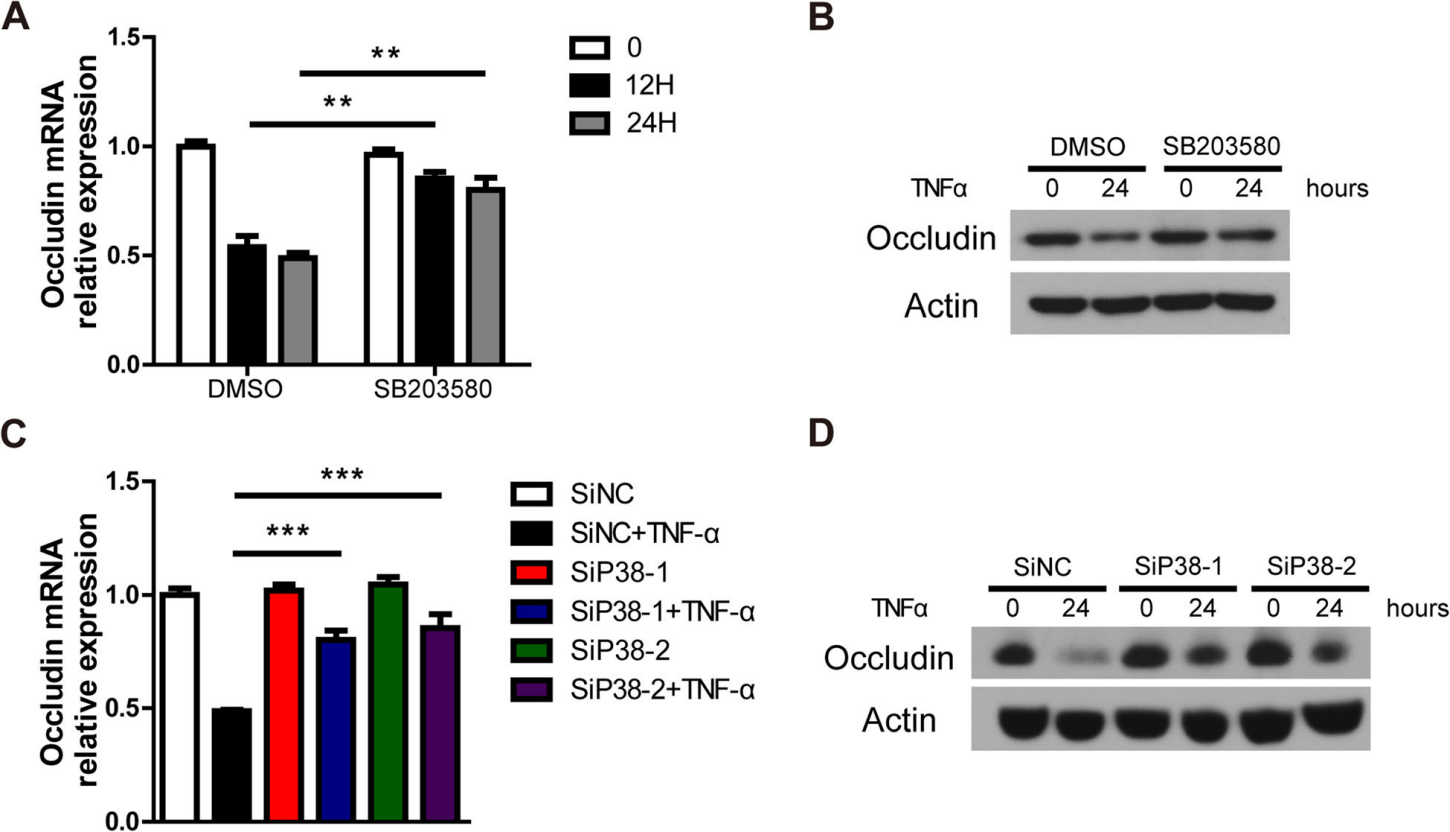
SB203580 or loss of P38 suppressed the decrease of Occludin expression in PIECs exposed to TNF-α. **a** PIECs were pretreated with DMSO, or SB203580 for 30 min and then exposed to rhTNF-α (20 ng/ml) for 0, 12, or 24 h. Total RNA was collected and the mRNA level of Occludin was measured by RT-PCR. **b** PIECs were pretreated with DMSO, or SB203580 for 30 min and then exposed to rhTNF-α (20 ng/ml) for 0, or 24 h. Lysates were analyzed by immunoblotting with antibodies against Occludin and actin. **c** The siP38 oligos and control siRNA oligo were transfected into PIECs. After 48 h, the transfected cells were treated with or without rhTNF-α (20 ng/ml) for 24 h. Total RNA was collected and the mRNA level of Occludin was measured by RT-PCR. **d** The siP38 oligos and control siRNA oligo were transfected into PIECs. After 48 h, the transfected cells were treated with rhTNF-α (20 ng/ml) for 0, or 24 h. Lysates were analyzed by immunoblotting with antibodies against Occludin and actin. Data are representative of at least three independent experiments (mean ± SEM). **p < 0.01, ***p < 0.001 by Student’s t test

According to the above findings, we predicted that TNF-α-decreased Occludin expression was dependent on P38. To prove our hypothesis, we inhibited P38 signaling pathway with P38 inhibitor or P38-specific siRNA oligos in PIECs exposed to TNF-α, and checked Occludin expression with real-time PCR and western-blotting assay. The mRNA level of Occludin was significantly increased in the SB203580 + TNF-α group compared to the DMSO+TNF-α group (Fig. 7a). We obtained a similar result of the protein level by western-blotting assay (Fig. 7b). Loss of P38 with P38-specific siRNA oligos obviously suppressed TNF-α-mediated reduction of Occludin mRNA or protein level (Fig. 7c-d). The data suggest that P38 signaling pathway is required for the TNF-α-mediated reduction of Occludin expression.

### Increased permeability might not be the prime reason for the augmented cytotoxicity of PIECs by TNF-α

**Fig. 8:**
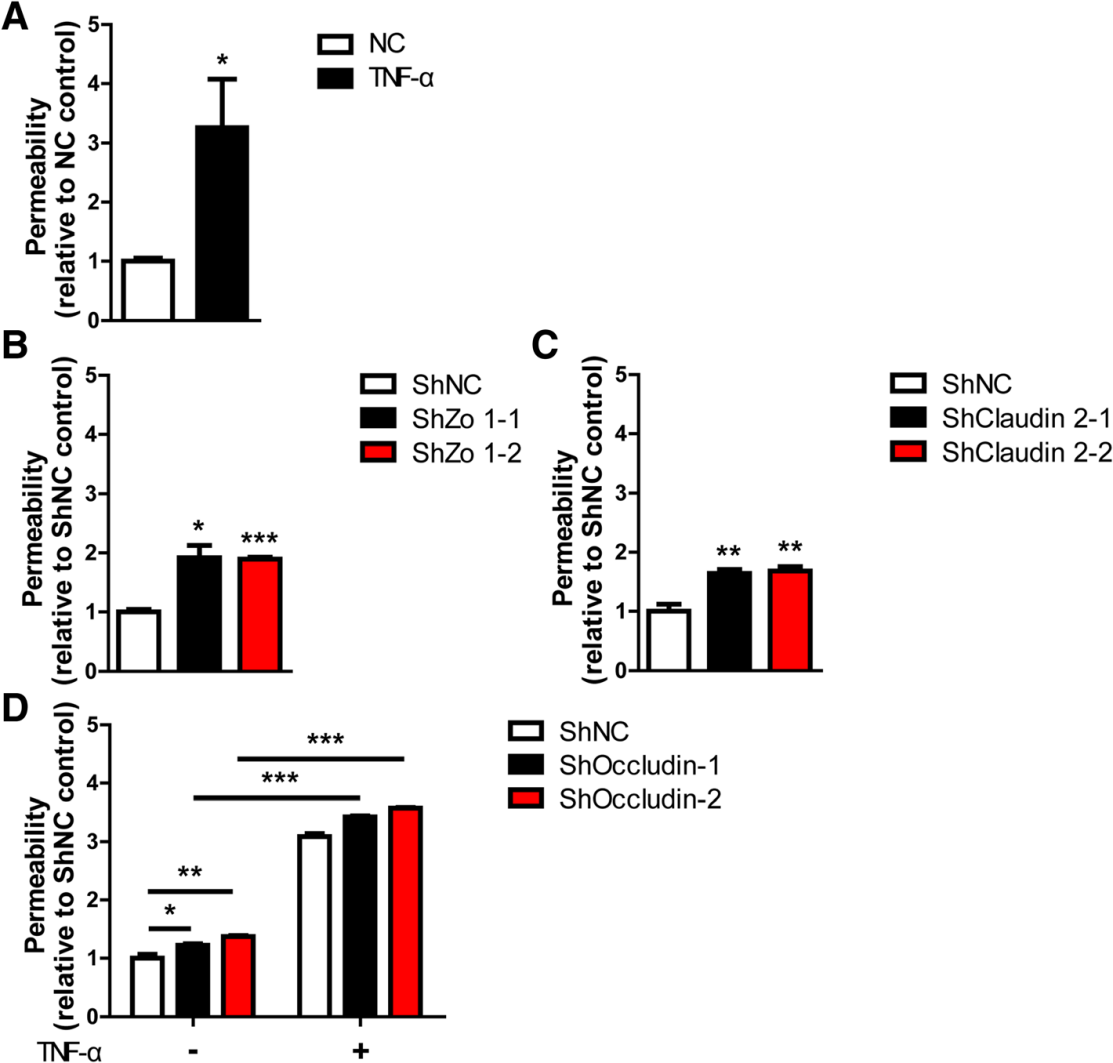
TNF-α increased the permeability of control PIECs and Occludin gene- silencing PIECs. **a** PIECs were treated with or without rhTNF-α (20 ng/ml) for 48 h. The permeability of a treated cell monolayer was assessed with transendothelial flux of FITC-Dextran. **b-c** PIECs were infected with control lentivirus or lentivirus expressing Zo 1- (**b**), or Claudin 2- (**c**) specific siRNA. The permeability of an infected cell monolayer was assessed by transendothelial flux of FITC-Dextran. **d** PIECs were infected with control lentivirus or lentivirus expressing Occludin-specific siRNA, and then treated with or without rhTNF-α (20 ng/ml) for 48 h. The permeability of an infected cell monolayer was assessed by transendothelial flux of FITC-Dextran. Data are representative of at least three independent experiments (mean ± SEM). *p < 0.05, **p < 0.01, ***p < 0.001 by Student’s t test

Occludin is an important tight junction protein which plays a critical role in maintaining barrier function [24–26]. Moreover, several reports demonstrated that TNF-α decreased barrier function by increasing permeability in epithelial and endothelial cells [27–30]. We asked whether TNF-α augmented the cytotoxicity of PIECs by increasing the permeability of PIECs. A permeability assay showed that TNF-α increased permeability about 3-fold compared to controls (Fig. 8a). Loss of Zo 1 or Claudin 2 increased the permeability of PIECs (Fig. 8b,c). However, loss of Zo 1 had no effect on the cytotoxicity of PIECs, and the loss of Claudin 2 group decreased the cytotoxicity of PIECs compared to controls (Fig. 5d,e). Loss of Occludin also augmented the permeability of PIECs (Fig. 8d). TNF-α also greatly increased the permeability in the Occludin-knockdown group (Fig. 8d). Additionally, the permeability of the TNF-α-treated Occludin-knockdown group was a little higher than in the TNF-α-treated control group (Fig. 8d).

Collectively, TNF-α and Occludin knockdown increased the permeability of PIECs, though this might not be the primary reason for the TNF-α-augmented cytotoxicity of PIECs.

## Discussion

Pro-inflammatory cytokines play important roles in immune regulation. Many diseases and allotransplantation are associated with cytokine dysregulation [31–33]. However, the role of cytokines in xenotransplantation has been less well investigated. In the current study, we found that human TNF-α significantly increased the cytotoxicity of porcine ECs in a human antibody-mediated CDC model. The mechanism study suggested that Occludin was essential for the TNF-α promotion of the killing of PIECs and TNF-α-augmented cytotoxicity of PIECs was largely suppressed by inhibition of P38. These findings demonstrated that TNF-α promoted the cytotoxicity of porcine ECs through downregulating the expression of Occludin, and this was dependent on the P38 signaling pathway. Our present study showed a novel pathological role of TNF-α in xenotransplantation.

Previously, we also found that human (and porcine) TNF-α induced the expression of pro-inflammatory genes and a pro-coagulation gene (tissue factor) in porcine (or human) ECs [15, 34]. Based on our observations, we confirm that TNF-α is likely to play potential pathological roles in xenotransplantation from various aspects. Blockade of TNF-α will probably be beneficial for the success of xenotransplantation.

Blockade of TNF-α in xenotransplantation has been reported by several groups [35–38]. Neutralizing antibody or etanercept (a TNF-α antagonist) has been a common way to inhibit TNF-α signaling pathway. Overexpression of soluble tumor necrosis factor-α receptor type I with human IgG1 Fc (sTNF-αR-Fc) in a xenograft, such as porcine islets or cells, or production of a transgenic sTNF-αR-Fc pig is another way to block TNF-α in xenotransplantation. Inhibition of TNF-α with some small molecule drugs may also be a promising approach.

Complement regulators (CD46, CD55 and CD59) are important negative regulators that restrain the over-activation of complement [21]. In the present study, we found that TNF-α increased the expression of complement regulators (CD46, CD55 and CD59), but did not affect complement activation. Cross-species incompatibilities might account for this. Antibody binding, C3 and C5b-9 deposition were also not modified by TNF-α. These data suggested that TNF-α increased the killing of PIECs, but not through promoting complement activation, MAC formation, or enhancing antibody binding.

Dalmasso et al. reported that IL-4 protected porcine ECs from human antibody-mediated CDC partially through upregulating the junction protein, Claudin 5, that strengthened the barrier function of porcine ECs [22]. We found that TNF-α decreased barrier function of PIECs. Loss of Occludin, Zo 1, and Claudin 5 increased cell permeability and decreased the barrier function of PIECs. However, loss of these three junction proteins had different effects on PIECs in the human antibody-mediated CDC model. Additionally, TNF-α largely enhanced the permeability of Occludin knockdown PIECs, but loss of Occludin obviously suppressed the enhanced cytotoxicity of PIECs treated by TNF-α. These findings demonstrated that decreased barrier function might not be the reason for the TNF-α promotion of killing of PIECs.

Collectively, our data demonstrated a novel function of Occludin which protected ECs from human antibody-mediated CDC. Our findings suggested that TNF-α promoted the killing of PIECs through regulating the expression of Occludin. We found that antibody binding, complement activation, MAC formation, or barrier disruption was not the major reason for TNF-α’s enhancement of cytotoxicity of PIECs. Based on these observations, we predicted that Occludin-mediated protection of the cells from human antibody-mediated CDC was likely associated with protection of the cells from MAC-mediated cytolysis. The inward leakage of water and ions, particularly a large influx of Ca^2+^ ions, is thought to be the prime reason for MAC-induced cell death [39]. Occludin might protect the cells in several possible ways - i) Since Occludin is a transmembrane protein, it might suppress the influx of Ca^2+^ ions or other ions by interaction with MAC; ii) Cell ion pumps put excess ions out of the cell, and Occludin might promote this through interacting with ion pumps; iii) Since ion pumps put the ions out of the cell, which requires energy, Occludin may be involved in energy metabolism. These hypotheses need to be investigated in the future.

Several papers have reported that TNF-α regulated the expression of Occludin [27–29, 40]. In different conditions, the regulation of Occludin by TNF-α is different. Wachtel et al. found that TNF-α decreased the expression of Occludin in mice astrocytes while TNF-α did not affect the expression of Occludin in human brain ECs, rat brain ECs (GP8.3), and MDCK epithelial cells [40]. Other groups found that Occludin was downregulated by TNF-α in human ECs (hCMEC/D3 and HUVECs) [27, 29]. Surprisingly, TNF-α increased the expression of Occludin in bovine retinal ECs (BRECs) [28].

In our setting, TNF-α decreased the expression of Occludin at mRNA and protein levels. However, how TNF-α-mediated the reduction of Occludin protein expression is an interesting scientific question. MAPKs (P38, JNK, and ERK) are intracellular signaling pathways that play a pivotal role in many complex cellular programs, such as cell proliferation, differentiation, development, and survival [41, 42]. Here, we found that JNK and ERK were not required for TNF-α-mediated reduction of Occludin expression. Interestingly, the TNF-α-induced decrease of Occludin was largely reversed by SB203580. Both the mRNA and protein levels of Occludin were reversed by SB203580. We also found that loss of P38 using siRNA oligos significantly suppressed TNF-α-induced Occludin mRNA or protein reduction. The data suggested that the TNF-α-decreased expression of Occludin was dependent on the P38 signaling pathway.

Here, we found that P38 siganling pathway was essential for TNF-α-mediated Occludin reduction. Tai et al. reported that amyloid-β induced Occludin down-regulation dependent on P38 activation in human brain ECs [43]. CdCl_2_-induced Occludin loss was also reversed by SB202190 (a specific P38 inhibitor) in rats [44]. However, how P38 regulated the expression of Occludin is not clear. Reduction of both mRNA and protein levels of Occludin by TNF-α was reversed by SB203580 or siRNA oligos targeting P38. The data suggested that P38 regulated the mRNA transcription of Occludin. In summary, TNF-α activated P38, the activated P38 entered into the cell nucleus and then phosphorylated downstream genes which bound to the promoter of Occludin and regulated its expression. Since P38 could activate many downstream genes, which genes are involved in Occludin expression needed to be further investigated.

Besides the transcription level, P38 might regulate the protein level of Occludin directly. Several papers reported that Occludin served as a substrate for matrix metalloproteinases 9 (MMP9) [45, 46]. TNF-α-induced MMP9 expression is dependent on P38 signaling in human urinary bladder cancer 5637 cells or human monocytes [47, 48]. We predicted that TNF-α-induced Occludin reduction was probably through MMP9-mediated proteolytic degradation, and P38 was required for TNF-α-induced MMP9 production. We will verify the hypothesis in the near future.

In the present study, we found that retrovirus-mediated ectopic expression of Occludin in PIECs protected the PIECs from human antibody-mediated CDC. The data suggested that in vivo gene transfer might be a promising way to protect porcine cells, tissues, or organs from the complement-mediated injury. However, we need more evidence before we overexpress Occludin in animal models of xenotransplantation. We are also investigating whether Occludin affects other important aspects of xenotransplantation, such as coagulation and inflammation. If the data suggest that Occludin indeed mediates the protection of xenografts, Occludin-transgenic pigs may need to be put on the agenda. Retroviruses are not suitable for clinical application, and are known to cause oncogenesis [49]. Use of adenovirus associated virus (AAV) might be a promising alternative approach. Interestingly, we found that, using lentivirus, loss of Claudin 2 in PIECs induced protection against human antibody-mediated CDC, which was the opposite effect to Claudin 5 and Occludin. The molecular mechanism is worthy of further investigation.

## Conclusions

In conclusion, in the present study, we found that human TNF-α promoted the cytotoxicity of PIECs by decreasing the expression of Occludin. The P38 signaling pathway was essential for TNF-α to mediate the reduction of Occludin expression and to increase human antibody-mediated CDC. Our study provides a possible explanation for the pathological role of TNF-α in xenotransplantation. In pig-to-human xenotransplantation, we suggest that blockade of TNF-α would probably decrease xenograft rejection and increase graft survival.

## Additional files

Additional file 1: **Table S1.** Listing of primers and primer sequences for real-time PCR. (DOC 47 kb)

Additional file 2: **Figure S1.** TNF-α did not cause PIEC death. **(A)** PIECs were treated with or without rhTNF-α (20 ng/ml) for 48 h, rhTNF-α (20 ng/ml) + cycloheximide (CHX, High: 10 μg/ml) or rhTNF-α (20 ng/ml) + CHX (Low: 2 μg/ml) for 2.5 h as positive controls. Lysates were analyzed by western-blotting with antibodies against Cleaved Caspase 3 and actin. **(B)** PIECs were treated with rhTNF-α (20 ng/ml) or medium as a negative control (NC) for 0, 24 h, 48 h, 72 h, or 96 h. The cell number was assessed with CCK8. **(C)** PIECs were treated with recombinant human IL-2 (20 ng/ml), IL-15 (100 ng/ml), IL-8 (100 ng/ml), IL-6 (20 ng/ml), G-CSF (100 ng/ml), G-MCSF (50 ng/ml), IFNγ (50 ng/ml), IL-4 (20 ng/ml), or medium as a negative control (NC) for 48 h. The cell number was assessed with CCK8. Data are representative of at least three independent experiments (mean ± SEM). *p < 0.05 by Student’s t test. L.E. = long exposure, S.E. = short exposure. (DOC 147 kb)

Additional file 3: **Figure S2.** Occludin did not affect expression of other junction genes in PIECs. **(A)** PIECs were infected with control virus (EV) or retrovirus encoding Occludin. After 4 days, total RNA was collected and the mRNA levels of indicated genes were measured by RT-PCR. **(B-C)** PIECs were infected with control lentivirus or with lentivirus expressing Occludin- (**B**) or Claudin 2- (**C**) specific siRNA. After 4 days, total RNA was collected and the mRNA levels of indicated genes were measured by RT-PCR. Data are representative of at least three independent experiments (mean ± SEM). *p < 0.05, **p < 0.01 by Student’s t test. (DOC 287 kb)

Additional file 4: **Figure S3.** Loss of P38 in PIECs was mediated by P38-specific siRNA oligos. **(A-B)** PIECs were transfected with control siRNA oligo or with P38-specific siRNA oligos. After 48 h, total RNA was collected and the mRNA levels of P38 were measured by RT-PCR (**A**), and lysates were analyzed by western-blotting (right side) with antibodies against P38, or actin (**B**). Data are representative of at least three independent experiments (mean ± SEM). ***p < 0.001 by Student’s t test. (DOC 98 kb)

## Data Availability

All data generated in this study are included in the manuscript.

## Abbreviations

ADV: Adenovirus
CDC: Complement-dependent cytotoxicity
EV: Empty vector
MAC: Membrane attack complex
MAPKs: Mitogen-activated protein kinases
PAECs: Porcine aortic endothelial cells
PIECs: Porcine iliac endothelial cells
TNF-α: Tumor necrosis factor-alpha
