# Supplementary material for: TNF-α promotes human antibody-mediated complement-dependent cytotoxicity of porcine endothelial cells through downregulating P38-mediated Occludin expression
Source: Cell Commun Signal. 2019 Jul 15;17:75. doi: 10.1186/s12964-019-0386-7 (PMC6631523; doi:10.1186/s12964-019-0386-7)
Supplement: Supplementary file 1 — Table S1. Listing of primers and primer sequences for real-time PCR. (DOC 47 kb) [file 12964_2019_386_MOESM1_ESM.doc]

**Table S1: Listing of primers and primer sequences for real-time PCR**

| **Gene name** | **Primers sequence (5’-3’)** |
| --- | --- |
| Porcine CD46 | acaccaatagccataaggatg |
|  | tccatttgggactactggat |
| Porcine CD55 | gcagactcagtgctctgtct |
|  | ggcactcatattccacggtg |
| Porcine CD59 | ttaggtcacagcctgcagtg |
|  | ggcacggcttcaacgaagat |
| Porcine Claudin 5 | tgggtaggcctgatcctg |
|  | cccttccaagtggtctgc |
| Porcine Occludin | ttatgcacccagcaacgac |
|  | ctggctgagaaagcattggt |
| Porcine Zo 1 | gagagccgggtctgagcta |
|  | aggtctctgctggcctgtc |
| Porcine Claudin 2 | gctggcgaacgagttcttac |
|  | ccttggagaagccgactg |
| Porcine Claudin 1 | ctccgcgcagtaacttcc |
|  | aaggcggagtttgcaggt |
| Porcine JAMA | tggttcaaggatggggtatt |
|  | gttgatgaaggcacggttg |
| Porcine PECAM1 | aaggtggagtcgtgaaggtc |
|  | tgaaatgtactggaggtttttcc |
| Porcine ESAM | ggctgggtcagtcctcttg |
|  | atcattcgctggctcctcta |
| Porcine CTNNB1 | tgctgttttgttccgaatgt |
|  | caaccgaaagccgtttctta |
| Porcine ICAM-1 | GAGGAGCTGTTCAGGCAGTC |
|  | CATCCGGAACGTGACATTG |
| Porcine VCAM-1 | CATTCCATGGTGTCCCAGA |
|  | TCCAAACTCTTCGTTTCCTTG |
| Porcine GAPDH | ACAGACAGCCGTGTGTTCC |
|  | ACCTTCACCATCGTGTCTCA |
| Human TNF-α | GAGGCCAAGCCCTGGTATG |
|  | CGGGCCGATTGATCTCAGC |
