# Supplementary material for: TNF-α promotes human antibody-mediated complement-dependent cytotoxicity of porcine endothelial cells through downregulating P38-mediated Occludin expression
Source: Cell Commun Signal. 2019 Jul 15;17:75. doi: 10.1186/s12964-019-0386-7 (PMC6631523; doi:10.1186/s12964-019-0386-7)
Supplement: Supplementary file 2 — Figure S1. TNF-α did not cause PIEC death. (A) PIECs were treated with or without rhTNF-α (20 ng/ml) for 48 h, rhTNF-α (20 ng/ml) + cycloheximide (CHX, High: 10 μg/ml) or rhTNF-α (20 ng/ml) + CHX (Low: 2 μg/ml) for 2.5 h as positive controls. Lysates were analyzed by western-blotting with antibodies against Cleaved Caspase 3 and actin. (B) PIECs were treated with rhTNF-α (20 ng/ml) or medium as a negative control (NC) for 0, 24 h, 48 h, 72 h, or 96 h. The cell number was assessed with CCK8. (C) PIECs were treated with recombinant human IL-2 (20 ng/ml), IL-15 (100 ng/ml), IL-8 (100 ng/ml), IL-6 (20 ng/ml), G-CSF (100 ng/ml), G-MCSF (50 ng/ml), IFNγ (50 ng/ml), IL-4 (20 ng/ml), or medium as a negative control (NC) for 48 h. The cell number was assessed with CCK8. Data are representative of at least three independent experiments (mean ± SEM). *p < 0.05 by Student’s t test. L.E. = long exposure, S.E. = short exposure. (DOC 147 kb) [file 12964_2019_386_MOESM2_ESM.doc]

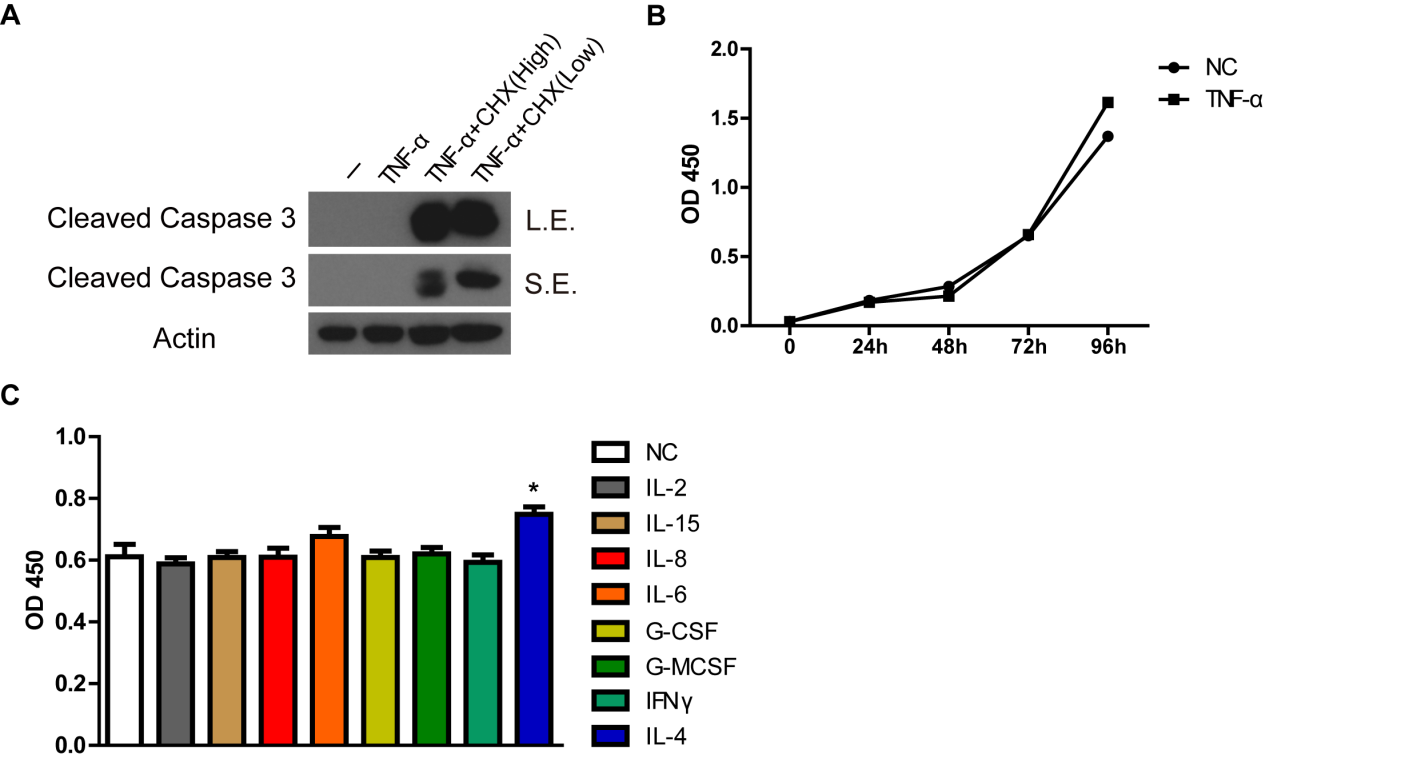


**Figure S1. TNF-α did not cause PIEC death.** **(A)** PIECs were treated with or without rhTNF-α (20 ng/ml) for 48h, rhTNF-α (20 ng/ml) + cycloheximide **(**CHX, High: 10 μg/ml) or rhTNF-α (20 ng/ml) + CHX (Low: 2 μg/ml) for 2.5h as positive controls. Lysates were analyzed by western-blotting with antibodies against Cleaved Caspase 3 and actin. **(B)** PIECs were treated with rhTNF-α (20 ng/ml) or medium as a negative control (NC) for 0, 24h, 48h, 72h, or 96h. The cell number was assessed with CCK8. **(C)** PIECs were treated with recombinant human IL-2 (20 ng/ml), IL-15 (100 ng/ml), IL-8 (100 ng/ml), IL-6 (20 ng/ml), G-CSF (100 ng/ml), G-MCSF (50 ng/ml), IFNγ (50 ng/ml), IL-4 (20 ng/ml), or medium as a negative control (NC) for 48h. The cell number was assessed with CCK8. Data are representative of at least three independent experiments (mean±SEM). **p* < 0.05 by Student’s t test. L.E.=long exposure, S.E.=short exposure.
