# Supplementary material for: TNF-α promotes human antibody-mediated complement-dependent cytotoxicity of porcine endothelial cells through downregulating P38-mediated Occludin expression
Source: Cell Commun Signal. 2019 Jul 15;17:75. doi: 10.1186/s12964-019-0386-7 (PMC6631523; doi:10.1186/s12964-019-0386-7)
Supplement: Supplementary file 3 — Figure S2. Occludin did not affect expression of other junction genes in PIECs. (A) PIECs were infected with control virus (EV) or retrovirus encoding Occludin. After 4 days, total RNA was collected and the mRNA levels of indicated genes were measured by RT-PCR. (B-C) PIECs were infected with control lentivirus or with lentivirus expressing Occludin- (B) or Claudin 2- (C) specific siRNA. After 4 days, total RNA was collected and the mRNA levels of indicated genes were measured by RT-PCR. Data are representative of at least three independent experiments (mean ± SEM). *p < 0.05, **p < 0.01 by Student’s t test. (DOC 287 kb) [file 12964_2019_386_MOESM3_ESM.doc]

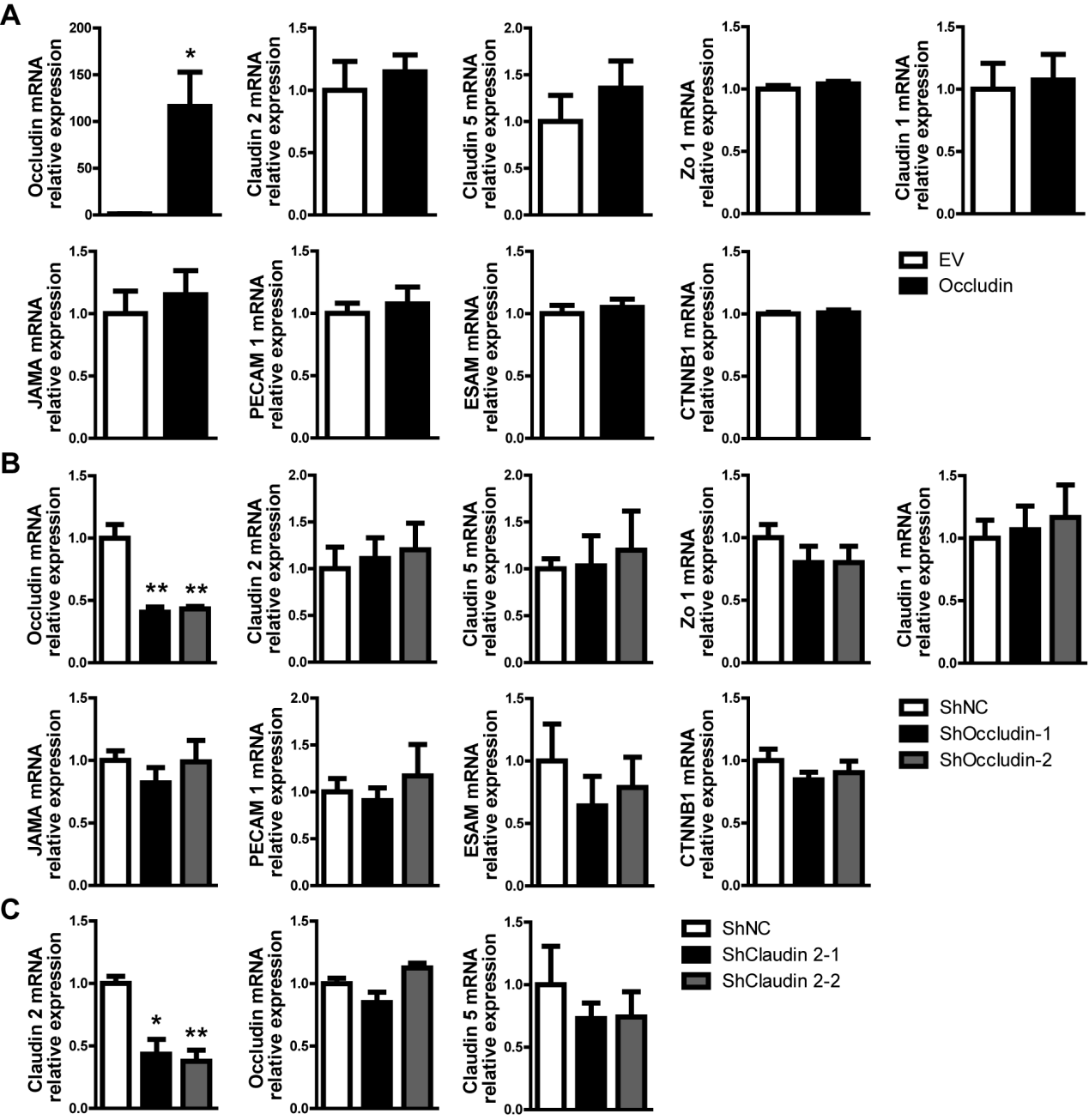


**Figure S2. Occludin did not affect expression of other junction genes in PIECs. (A)** PIECs were infected with control virus (EV) or retrovirus encoding Occludin. After 4 days, total RNA was collected and the mRNA levels of indicated genes were measured by RT-PCR. **(B-C)** PIECs were infected with control lentivirus or with lentivirus expressing Occludin- (**B**) or Claudin 2- (**C**) specific siRNA. After 4 days, total RNA was collected and the mRNA levels of indicated genes were measured by RT-PCR. Data are representative of at least three independent experiments (mean±SEM). **p* < 0.05, ***p* < 0.01 by Student’s t test.
