# Supplementary material for: TNF-α promotes human antibody-mediated complement-dependent cytotoxicity of porcine endothelial cells through downregulating P38-mediated Occludin expression
Source: Cell Commun Signal. 2019 Jul 15;17:75. doi: 10.1186/s12964-019-0386-7 (PMC6631523; doi:10.1186/s12964-019-0386-7)
Supplement: Supplementary file 4 — Figure S3. Loss of P38 in PIECs was mediated by P38-specific siRNA oligos. (A-B) PIECs were transfected with control siRNA oligo or with P38-specific siRNA oligos. After 48 h, total RNA was collected and the mRNA levels of P38 were measured by RT-PCR (A), and lysates were analyzed by western-blotting (right side) with antibodies against P38, or actin (B). Data are representative of at least three independent experiments (mean ± SEM). ***p < 0.001 by Student’s t test. (DOC 98 kb) [file 12964_2019_386_MOESM4_ESM.doc]

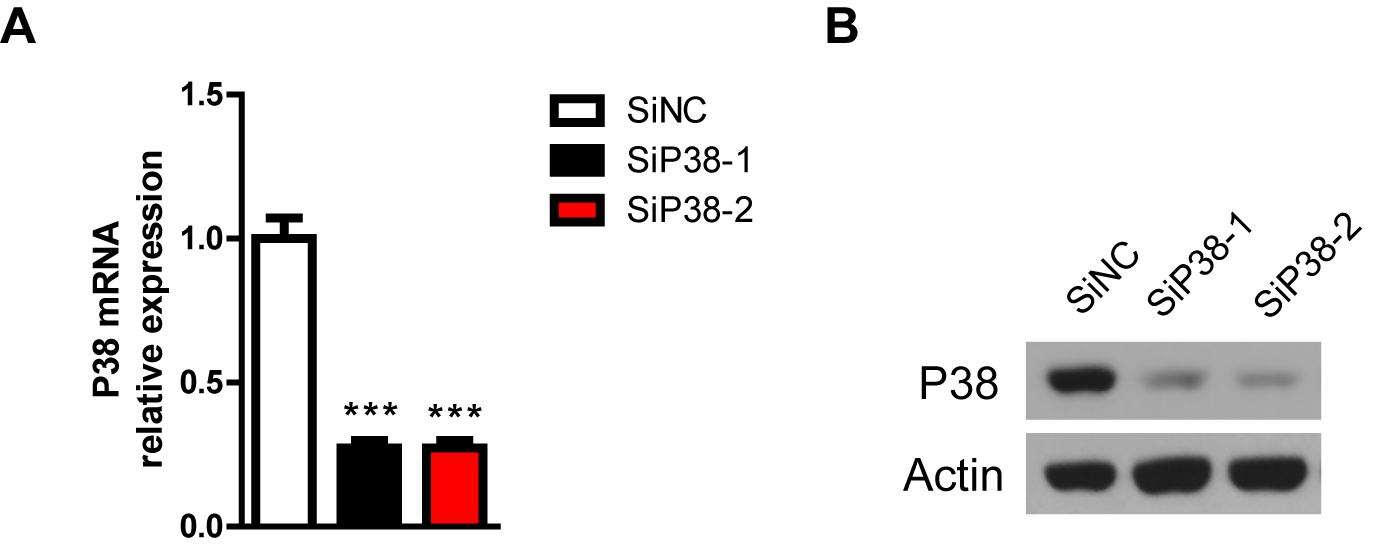


**Figure S3. Loss of P38 in PIECs was mediated by P38-specific siRNA oligos.** **(A-B)** PIECs were transfected with control siRNA oligo or with P38-specific siRNA oligos. After 48h, total RNA was collected and the mRNA levels of P38 were measured by RT-PCR (**A**), and lysates were analyzed by western-blotting (right side) with antibodies against P38, or actin (**B**). Data are representative of at least three independent experiments (mean±SEM). ****p* < 0.001 by Student’s t test.
